# Examination of the relationship between viscoelastic properties and the invasion of ovarian cancer cells by atomic force microscopy

**DOI:** 10.3762/bjnano.11.45

**Published:** 2020-04-06

**Authors:** Mengdan Chen, Jinshu Zeng, Weiwei Ruan, Zhenghong Zhang, Yuhua Wang, Shusen Xie, Zhengchao Wang, Hongqin Yang

**Affiliations:** 1 Key Laboratory of Optoelectronic Science and Technology for Medicine of Ministry of Education, Fujian Provincial Key Laboratory for Photonics Technology, Fujian Normal University, Fuzhou 350007, China; 2 Department of Ultrasound Medical, The First Affiliated Hospital of Fujian Medical University, Fuzhou 350005, China; 3 Fujian Provincial Key Laboratory for Developmental Biology and Neurosciences, College of Life Sciences, Fujian Normal University, Fuzhou 350007, China

**Keywords:** atomic force microscopy (AFM), cancer invasion, cancer migration, ovarian cancer cells, viscoelasticity

## Abstract

The mechanical properties of cells could serve as an indicator for disease progression and early cancer diagnosis. This study utilized atomic force microscopy (AFM) to measure the viscoelastic properties of ovarian cancer cells and then examined the association with the invasion of ovarian cancer at the level of living single cells. Elasticity and viscosity of the ovarian cancer cells OVCAR-3 and HO-8910 are significantly lower than those of the human ovarian surface epithelial cell (HOSEpiC) control. Further examination found a dramatic increase of migration/invasion and an obvious decease of microfilament density in OVCAR-3 and HO-8910 cells. Also, there was a significant relationship between viscoelastic and biological properties among these cells. In addition, the elasticity was significantly increased in OVCAR-3 and HO-8910 cells after the treatment with the anticancer compound echinomycin (Ech), while no obvious change was found in HOSEpiC cells after Ech treatment. Interestingly, Ech seemed to have no effect on the viscosity of the cells. Ech significantly inhibited the migration/invasion and significantly increased the microfilament density in OVCAR-3 and HO-8910 cells, which was significantly related with the elasticity of the cells. An increase of elasticity and a decrease of invasion were found in OVCAR-3 and HO-8910 cells after Ech treatment. Together, this study clearly demonstrated the association of viscoelastic properties with the invasion of ovarian cancer cells and shed a light on the biomechanical changes for early diagnosis of tumor transformation and progression at single-cell level.

## Introduction

Ovarian cancer is a lethal gynecological malignancy with low survival rates due to the fact that the disease is generally diagnosed during the late stages [1–2]. Discriminating more tumorigenic from less tumorigenic cancer cells contributes to the determination of disease severity and personalized treatment [3]. A close relationship between the progression of cancer and the change of mechanical properties of the cells has been discovered in the last decades [4–5]. Mechanical properties used to determine the tumorigenic and metastatic potential of cells are strongly associated with cell transformation, migration and invasion [6]. Therefore, diseased cells could be detected biomechanically.

At present, a variety of research technologies, such as optical tweezers, micropipette aspiration, magnetic twisting cytometry and atomic force microscopy (AFM), have been developed to characterize the mechanical properties of biological samples [7–10]. Among these, AFM is widely used because of the convenience of sample preparation and the ability to examine accurately mechanical properties. Studies using AFM have revealed that viscoelastic properties are novel indicators that can be used to differentiate cancerous from healthier cells [11].

Cell migration and invasion are the two key processes leading to the spread of cancer cells from primary tumors to distant organs during tumor metastasis [12–13]. They are largely related to cytoskeleton structure [14–15]. In addition, stiffness and deformation of cells are strongly regulated by the microfilament skeleton [16]. Therefore, the rearrangement of microfilament skeleton is crucial for cell motility and contributes largely to elasticity changes of the cells, when the cytoskeleton structure changes from a more organized to a disordered form with the transformation from benign to malignant [17–18]. However, the association of viscoelastic properties with the invasion of ovarian cancer cells is not well understood.

Chemotherapy is approved as the most effective treatment for advanced-stage ovarian cancer [19–23]. Only few studies focus on the variability in mechanical properties related with cancer invasion after anticancer drug treatment [24–25]. Echinomycin serves as a potential therapeutic agent through the induction of cell apoptosis, which is typically used in the treatment of epithelial cancers, including ovary, breast and prostate cancers [26–29]. Inhibitory mechanisms of cancer invasion and metastasis based on chemotherapy can be beneficial for both biomechanical research and clinical applications [30–31]. Therefore, the present study examined the elasticity and viscosity through AFM, and cell migration, invasion and microfilament density through cell experiments, and the relationship between them was established.

## Results and Discussion

### Viscoelastic properties of ovarian cancer cells

In the present study, the viscoelastic properties of ovarian cancer cells (OVCAR-3 and HO-8910) and human ovarian surface epithelial cells as control (HOSEpiC) were measured by AFM to discriminate normal ovarian cells from cancerous cells and more tumorigenic from less tumorigenic ovarian cancer cells. A previous study demonstrated the effect of indole-3-carbinol (I3C), indicating that I3C may inhibit metastasis and invasion of OVCAR-3 [32]. HBO1-directed histone H4 specific acetylation, potentiating the membrane elasticity of ovarian cancer cells, and HBO1 mRNA expression were significantly higher in OVCAR-3 cells than in the HOSEpiC control [33]. The different roles of lamin A and lamin C were also elucidated in the metastasis of ovarian cancer cells HO-8910 and SKOV-3. Therefore, the three cell lines OVCAR-3, HO-8910 and HOSEpiC were selected for the present work [34].

**Figure 1:**
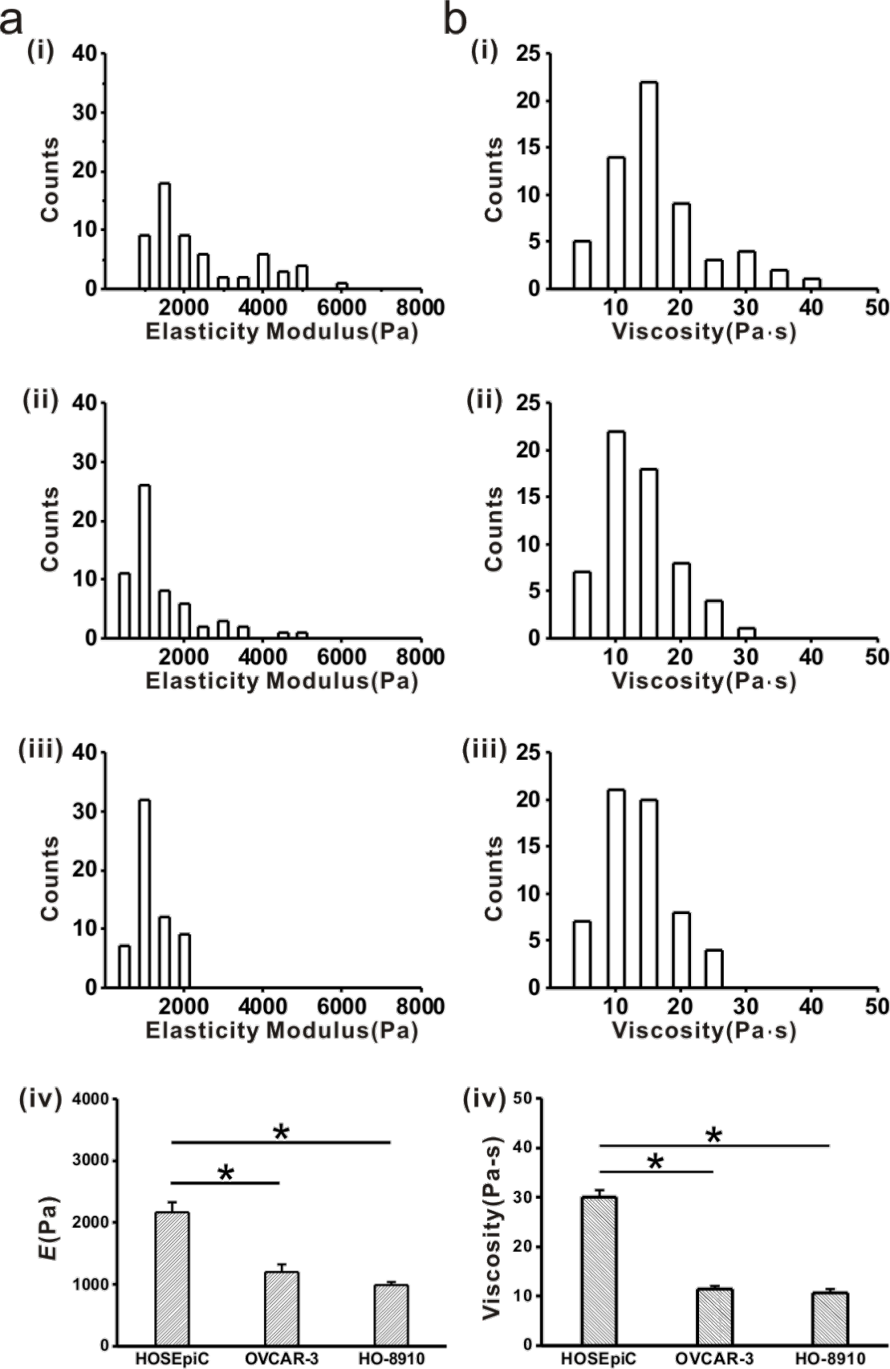
Histograms of viscoelastic properties of ovarian cells. Cell elasticity and viscosity were examined by atomic force microscopy. a-(i): Elastic modulus histogram of HOSEpiC cells, a-(ii): elastic modulus histogram of OVCAR-3 cells, a-(iii): elastic modulus histogram of HO-8910 cells; a-(iv): average elastic modulus values of ovarian cells. b-(i): Viscosity histogram of HOSEpiC cells, b-(ii): viscosity histogram of OVCAR-3 cells, a-(iii): viscosity histogram of HO-8910 cells, a-(iv) average viscosity values of ovarian cells. The data are presented as mean ± SE, and the asterisk indicates *p* < 0.05, *n* = 60.

The viscoelastic properties of cells include elasticity and viscosity, which are prominent biomechanical properties of cells. This study, the cell viscoelasticity was derived from the force indentation curves obtained with AFM on three different ovarian cell lines shown in Figure 1. The results of cell elasticity showed a concentrated and narrow distribution in OVCAR-3 and HO-8910 cells (Figure 1a-ii,iii) and a dispersive and broad distribution in HOSEpiC cells (Figure 1a-i). The average values of the elastic modulus of OVCAR-3 and HO-8910 cells were 1195.72 ± 122.94 Pa and 996.27 ± 52.56 Pa, respectively (Figure 1a-iv). These values are significantly lower than the elastic modulus of HOSEpiC cells (2160.94 ± 167.77 Pa, Figure 1a-iv), indicating the lower elasticity of ovarian cancer cells. The results are consistent with previous reports that the stiffness of normal cells is higher than that of breast cancer cells [35]. Therefore, the elasticity could be considered as an effective indicator to differentiate the state of tumor development.

Another important characteristic is viscosity, which reflects the viscoelastic response of cells to force stimulation [3], and the energy dissipated during the force indentation process [36]. The measurements of cell viscosity showed that the average values of OVCAR-3 (11.38 ± 0.72 Pa·s) and HO-8910 (10.70 ± 0.66 Pa·s) were significantly lower than that of HOSEpiC (30.00 ± 0.66 Pa·s, Figure 1b). Viscosity reflects the capabilities of motility and invasion of cells [37–38], and could be used to differentiate cancerous cells from the healthy cells [39–40]. Increased tumorigenic potential is associated with decreased cell viscosity, which has been demonstrated in previous studies [3].

### Tumorigenic properties of ovarian cancer cells

**Figure 2:**
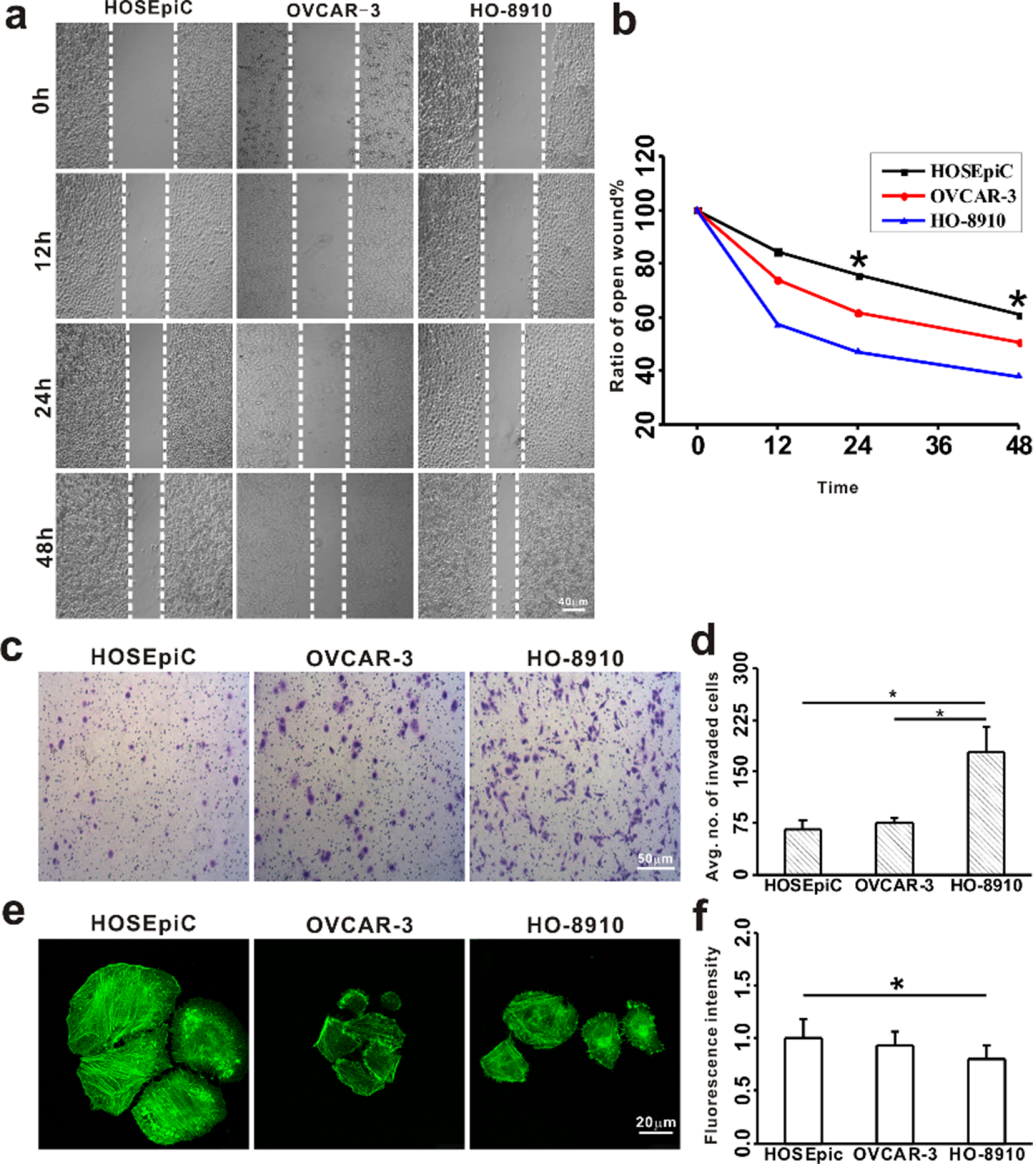
Analysis of tumorigenic properties of ovarian cancer cells. Migration and invasion of ovarian cancer cells were analyzed and the microfilament density was examined by imaging cytoskeleton F-actin. a: HO-8910, OVCAR-3 and HOSEpiC cells were cultured for 0 h, 12 h, 24 h and 48 h and the healing of cell scratches was observed; scale bar = 40 μm. b: The migration of ovarian cancer cells was calculated and the asterisk indicates *p* < 0.05. c: The invasion of ovarian cancer cells was examined; scale bar = 50 μm. d: The invasion of ovarian cancer cells was analyzed and the asterisk indicates *p* < 0.05. e: The microfilament density was examined by imaging cytoskeleton F-actin; scale bar = 20 μm. f: The microfilament density was analyzed and the asterisk indicates *p* < 0.05.

In order to determine the relationship between viscoelastic and tumorigenic properties of ovarian cancer cells, the present study further examined the migration and invasion changes of these cells besides the microfilament density of F-actin cytoskeleton. The migration of cancer cells is critical for their tumorigenic properties and can be measured by using cell migration assays [41]. The average healing rates of OVCAR-3 and HO-8910 were significantly greater than that of HOSEpiC (Figure 2a,b), which is consistent with the changes of viscoelastic results by AFM, indicating the relationship between migratory potential and viscoelastic properties of ovarian cancer cells. Zhou et al. found that Hey HM cells exhibited a higher migration capacity than NM cells, also indicating that the difference in stiffness in Hey A8 cells with different metastasis potential is related to changes in the cytoskeleton structure, which are similar with our results of this investigation [18]. Additionally, some studies have reported the cells with higher motility were usually associated with lower stiffness [42–43].

The invasion of ovarian cancer cells is another tumorigenic property and can be examined by using cell invasion assays [44]. The average numbers of invasive OVCAR-3 and HO-8910 cells were higher than the number of invasive HOSEpiC cells (Figure 2c), and HO-8910 cells had the highest invasion potential (Figure 2d). Xu’s group pointed out that ovarian cancer cells showed a lower elasticity than non-malignant ovarian epithelial cells. The increase of migratory and invasive capacity was also associated with a significant reduction of cellular elasticity. Similar to our findings, the elasticity and viscosity of ovarian cancer cells decreased, and the present study found an obvious increase of migration/invasion in cancer cells compared to the control cells [16]. The measured tumorigenic properties exhibit an inverse relationship to the cellular stiffness, the stiffness of cancer cells with higher invasive capability is lower than that of cells with lower invasive capability [45]. Furthermore, the relationship between invasiveness and viscoelastic properties was reviewed under biochemical modifications of cancer cells [46].

Furthermore, the microfilament density was examined by imaging cytoskeleton F-actin. ActinGreen (KeyGEN BioTECH) was used to investigate the distribution of the actin cytoskeleton among these cells. The density of actin filaments in HOSEpiC cells was higher than that in HO-8910 cells (Figure 2f), demonstrating the microfilament density is related to the viscoelsticity of the cells. This is also consistent with previous reports of cancer cells with a lower density of F-actin filament and a lower elasticity than normal cells [47–49].

### Correlation between viscoelastic and tumorigenic properties among ovarian cancer cells

The correlation between viscoelastic and tumorigenic properties was analyzed and further confirmed that the elastic properties are significantly related to the migration and invasion of ovarian cancer cells (Table 1). The initial AFM results have identified that OVCAR-3 and HO-8910 cells were much softer and more deformable than HOSEpiC cells. Gardel’s group also reported that the reduction of viscoelasticity was related with an increase of the migratory potential of cancer cells, providing a new understanding of the mechanisms in cancer development [8].

**Table 1:** Correlation analysis of elasticity and migration, invasion and F-actin density of three ovarian cell lines.

elasticity		HOSEpiC	OVCAR-3	HO-8910

migration	HOSEpiC	−0.732		
	OVCAR-3		−0.991	
	HO-8910			−0.9528
	
invasion	HOSEpiC	−0.788		
	OVCAR-3		−0.987	
	HO-8910			−0.990
	
F-actin density	HOSEpiC	0.963		
	OVCAR-3		0.932	
	HO-8910			0.941

The process of invasion and metastasis is based on the movement and deformation of cancer cells [8], and this process is related to the viscoelasticity of cells [36]. In the present AFM experiments, the elasticity and viscosity of OVCAR-3 and HO-8910 cells were lower than those of HOSEpiC cells, which further emphasized the role of viscoelastic properties in cell invasion. Therefore, analyzing the viscoelastic characteristics of cells contributes to further understanding the ability to deform and metastasize of cells, thus further predicting the development of cancer [50].

Given that the cytoskeleton plays a key role in the maintenance of cell morphology, mobility deformation and related information transmission become an important factor determining the mechanical properties of cells [50]. Some researchers have shown that alterations in the cytoskeletal structure or functional defects of cells are associated with the ability of tumor cells to proliferate [51]. The actin filaments on the cell membrane can affect the elasticity of the cells. The present results indicate that the microfilament density is related to the viscoelastic properties of ovarian cancer cells.

### Effects of the anticancer compound echinomycin on the viscoelastic properties of ovarian cells

**Figure 3:**
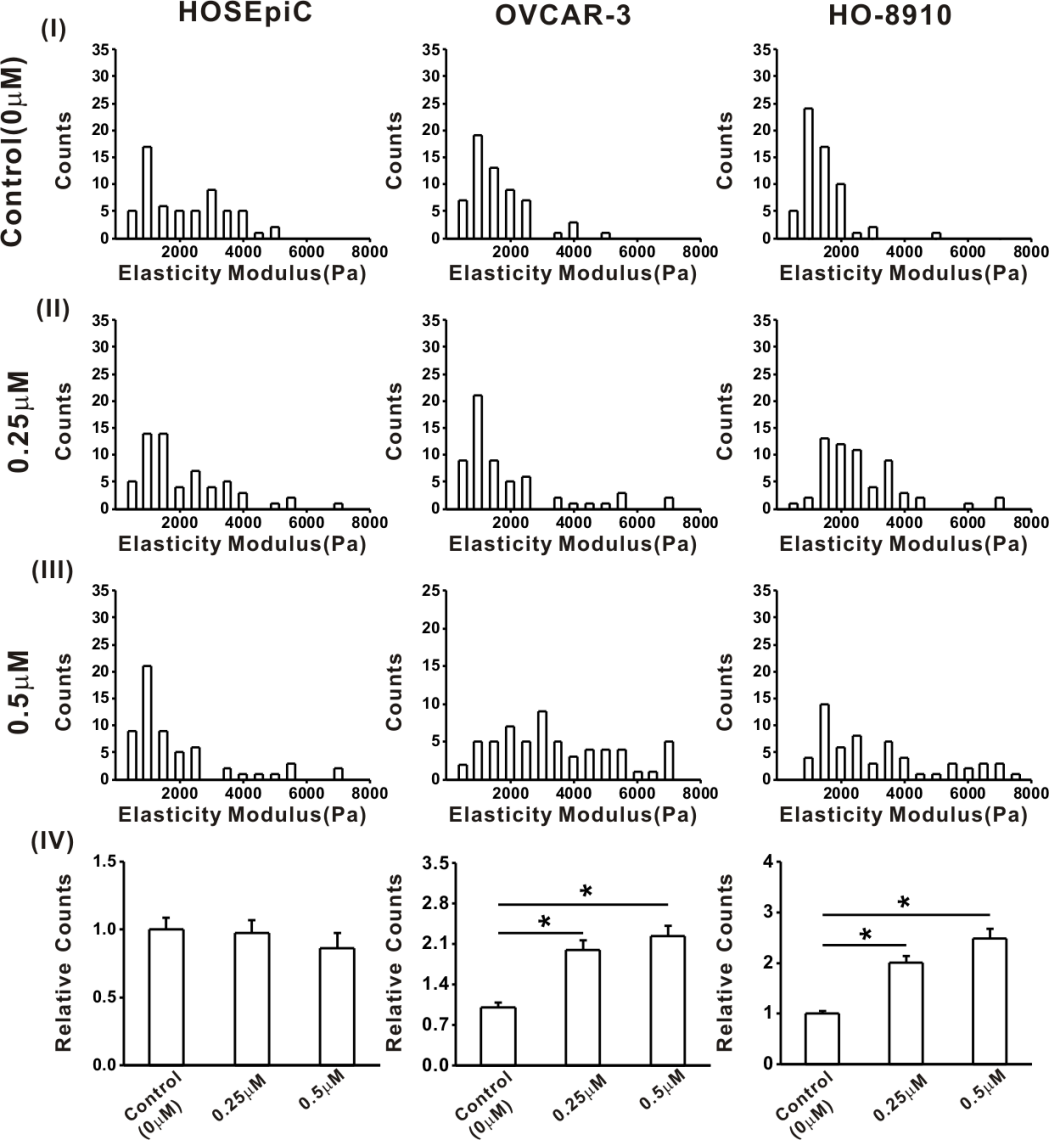
Histograms of the elastic modulus of ovarian cells. The HO-8910, OVCAR-3 and HOSEpiC cells were cultured and treated with 0 μM (control), 0.25 μM or 0.5 μM echinomycin. The data are presented as mean ± SE, and the asterisk indicates *p* < 0.05, *n* = 60.

**Figure 4:**
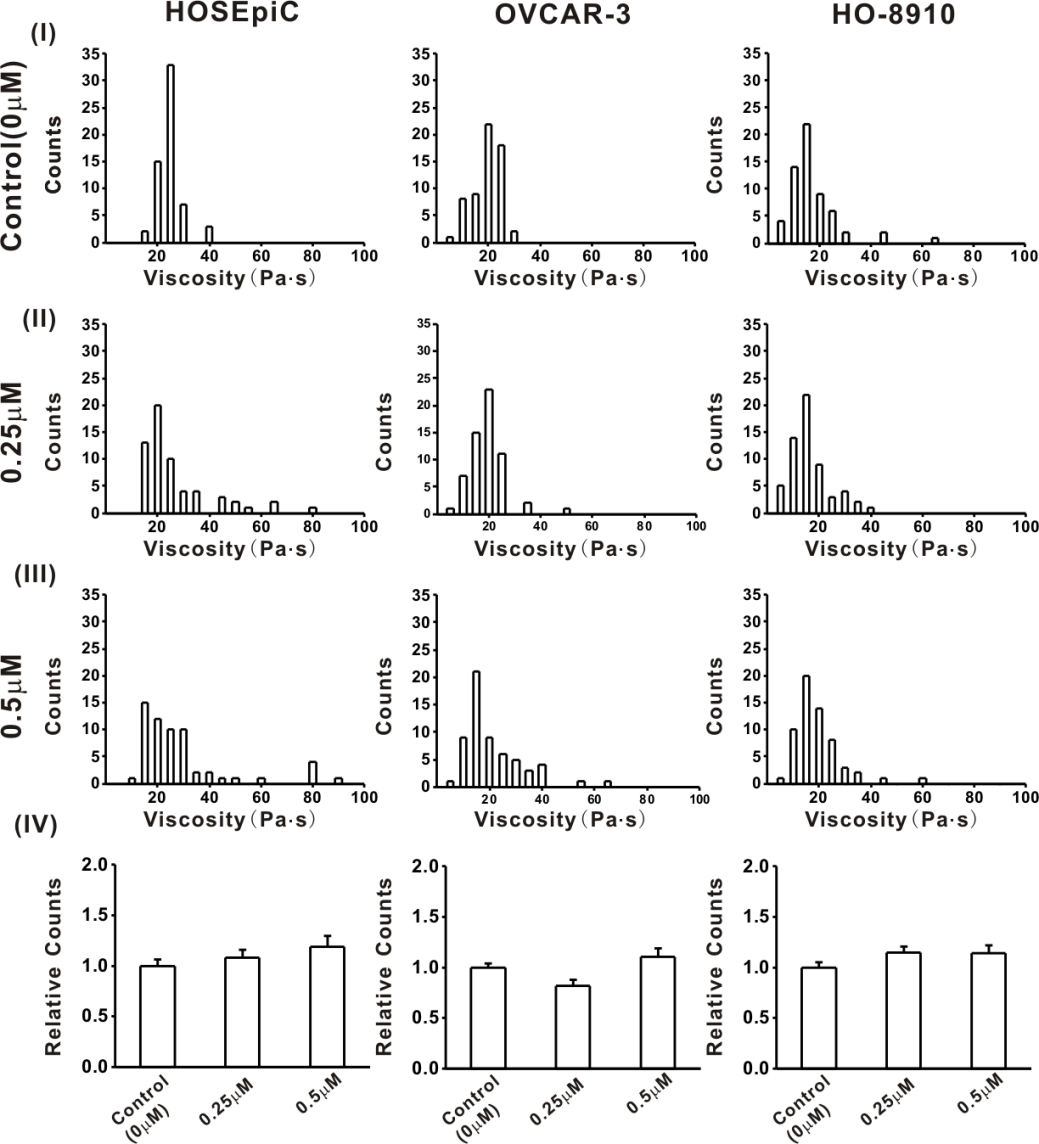
Histograms of the viscosity of ovarian cells. The HO-8910, OVCAR-3 and HOSEpiC cells were cultured and treated with 0 μM (control), 0.25 μM or 0.5 μM echinomycin. The data are presented as mean ± SE, and the asterisk indicates *p* < 0.05, *n* = 60.

For further identify the relationship between viscoelastic and tumorigenic properties, the cells were treated with the anticancer compound echinomycin (Ech) at different concentrations of 0, 0.25 and 0.5 μM for 3 h. Subsequently, the changes of cell viscoelasticity after Ech treatment were measured (Figure 3 and Figure 4). The results of cell elasticity showed the average elasticity of HO-8910 cells after exposure to 0.5 μM Ech for 3 h (2944.02 ± 238.88 Pa) was higher than that of the 0 μM control (1187.30 ± 54.27 Pa) and after treatment with 0.25 μM Ech (2377.22 ± 235.98 Pa, Figure 3). The average elasticity of OVCAR-3 cells treated with 0.25 μM Ech increased by approximately 57% compared to the control (Figure 3), while no obvious changes of the average elasticity was found among HOSEpiC cells (Figure 3). These findings demonstrated that the effect of Ech is related with an increased elasticity of ovarian cancer cells, which is consistent with a previous report that Ech induces alterations of the biomechanical properties of cancer cells [52]. The cell elasticity increased with the increase of the drug concentration, and the viscoelastic properties of cancer cells can be changed by antineoplastic drugs [53]. Our results are consistent with previous observations that drug stimulation caused mechanical stiffening of ovarian cancer cells [31]. Kim et al. showed the increased stiffness of highly metastatic human breast cancer cells after the activation of β-adrenergic signaling by βAR agonists, as well as an increased invasiveness of these cells in vitro [6]. These results were discussed in relation to the underlying mechanical mechanism of action in cancer cells [54].

The average viscosity values of HOSEpiC, OVCAR-3 and HO-8910 cells treated with 0.25 μM Ech for 3 h were 24.11 ± 1.81 Pa·s, 13.89 ± 1.03 Pa·s and 16.73 ± 0.89 Pa·s, respectively (Figure 4). After treatment with 0.5 μM Ech for 3 h, they changed to 26.6 ± 2.36 Pa·s, 18.72 ± 1.46 Pa·s and 16.6 ± 1.16 Pa·s, respectively (Figure 4). No obvious changes of the average viscosity of HOSEpiC, OVCAR-3 and HO-8910 cells were found after Ech treatment (Figure 4). Therefore, the detailed mechanisms related to the viscosity of ovarian cancer cells needs to be investigated further. The alterations of cell biomechanical properties after Ech treatment showed good agreement with the drug-mediated activation in both cells [55]. The results suggest that studying the changes of biomechanical properties of cancer cells using AFM could provide an important means for evaluating the anticancer activity of a drug [56].

### Effects of anticancer compound echinomycin on the tumorigenic properties of ovarian cancer cells

**Figure 5:**
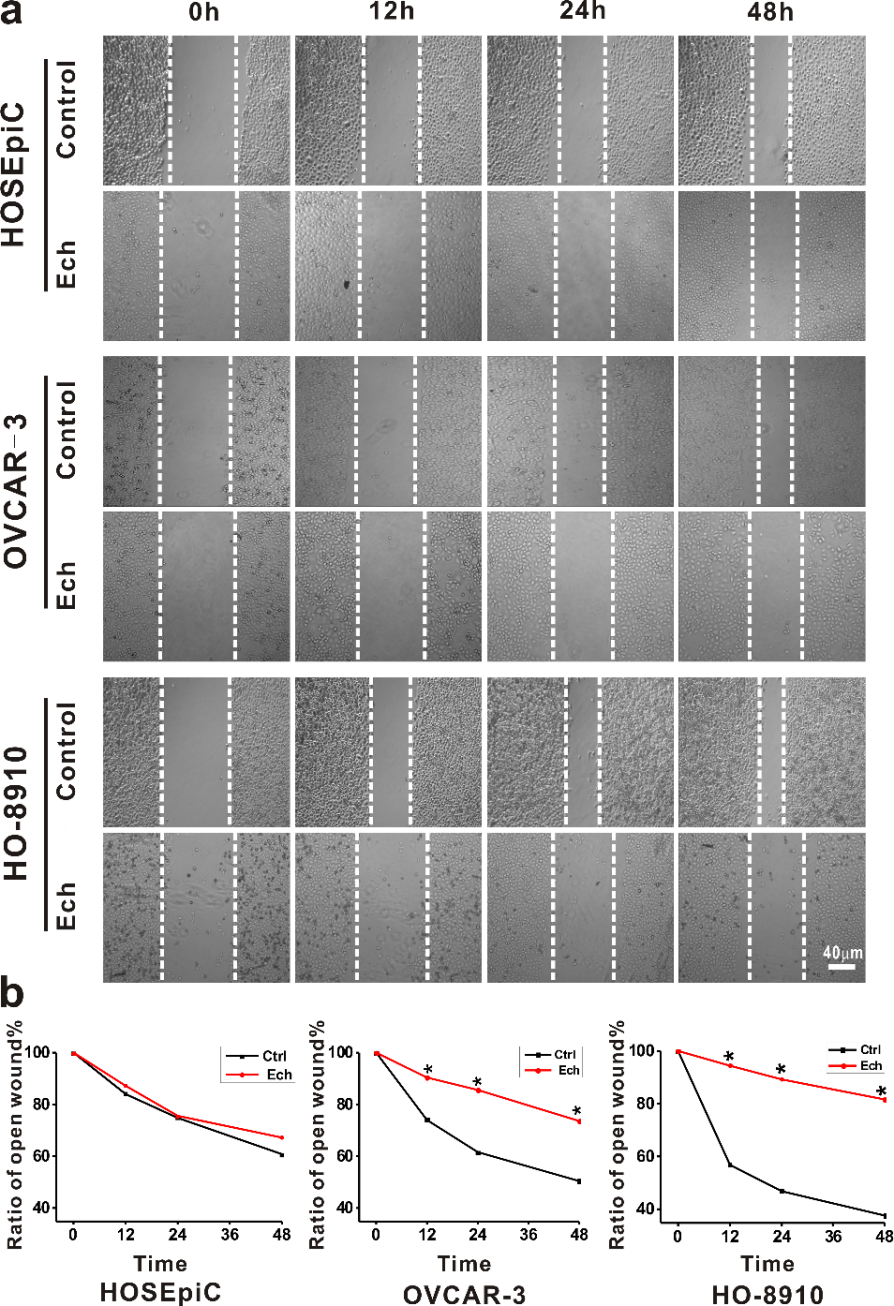
Migration analysis of ovarian cells treated with echinomycin. HO-8910, OVCAR-3 and HOSEpiC cells were cultured and treated with 0.25 μM echinomycin. a: The healing of cell scratches was observed after periods of time between 0 and 48 h; scale bar = 40 μm. b: The migration of ovarian cells was analyzed. The data are presented as mean ± SE, and the asterisk indicates *p* < 0.05.

**Figure 6:**
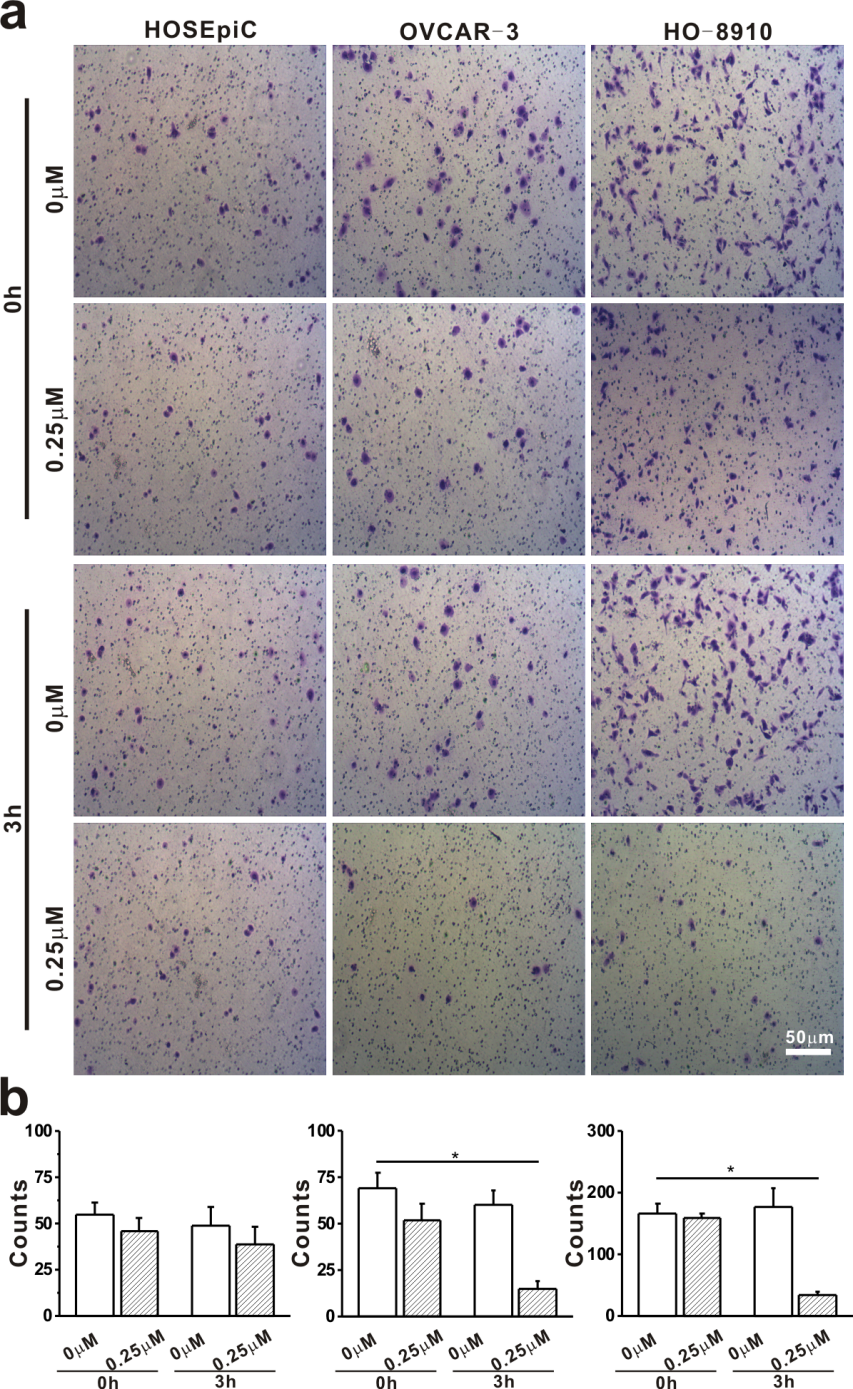
Invasion analysis of ovarian cells treated with echinomycin. HO-8910, OVCAR-3 and HOSEpiC cells were cultured and treated with 0.25 μM echinomycin for 3 h. a: Invasion of ovarian cells; scale bar = 50 μm. b: The invasion of ovarian cells was analyzed. The data are presented as mean ± SE, and the asterisk indicates *p* < 0.05.

**Figure 7:**
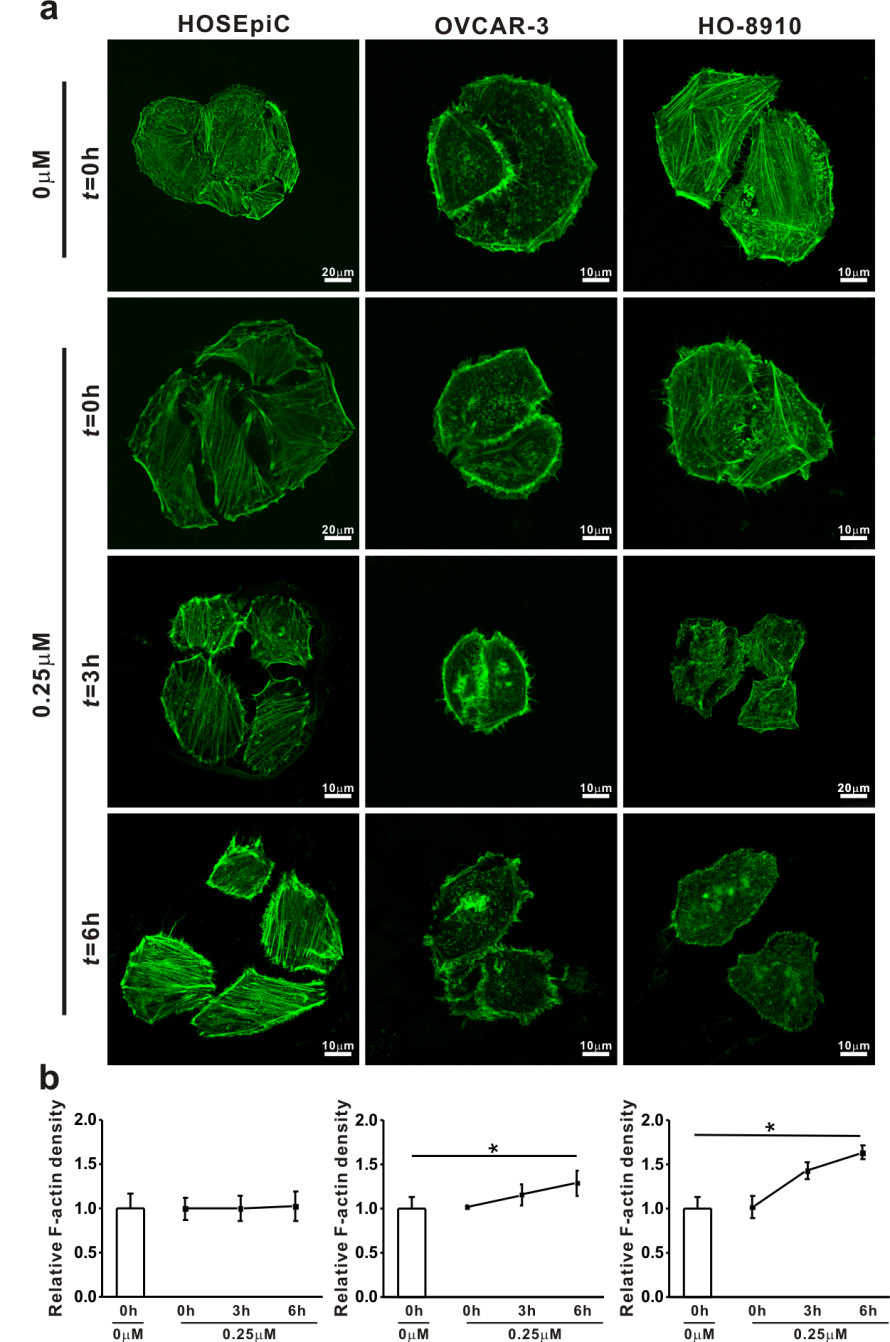
Analysis of the microfilament density through cytoskeleton F-actin imaging. HO-8910, OVCAR-3 and HOSEpiC cells were cultured and treated with 0.25 μM echinomycin for 3 h. a: Imaging of the cytoskeleton F-actin; scale bar = 20 μm. b: The microfilament density was analyzed. The data are presented as mean ± SE, and the asterisk indicates *p* < 0.05.

In order to clarify the effect of Ech on tumorigenic properties, the present study examined the changes in migration (Figure 5) and invasion (Figure 6), and the microfilament density of the F-actin cytoskeleton (Figure 7) of the cells after Ech treatment .

In the cell migration assay, the cells were scratched and treated with 0.25 μM echinomycin for 3 h, and then the cell migration was recorded after different periods of time. The present results showed that the migratory potential was inhibited after exposure to 0.25 μM Ech in OVCAR-3 and HO-8910 cells and not in HOSEpiC cells (Figure 5). These findings were consistent with previous reports that chemotherapy drugs could efficiently suppress the migration of cancer cells [57–58].

During cell invasion assay, the number of invasive HO-8910 and OVCAR-3 cells after treatment with 0.25 μM Ech for 3 h was much lower than that of the control, while no obvious changes were found in HOSEpiC cells (Figure 6), implying a greater impact of Ech on the invasive capacity of HO-8910 and OVCAR-3 than on that of HOSEpiC cells, which is consistent with the changes of AFM results. These results further illustrate that the mechanical properties of cells are related to their invasive potential. Cancer cells need the ability for deformation for migration and invasion [59]. Kinsella’s group showed that the main problem in the treatment of cancer may be the invasive behavior of cancer cells [60]. The present investigations indicate that chemotherapy drugs could alter the mechanical properties of malignant tumors cells to attenuate cell proliferation, migration and invasion [61]. Lian et al. have shown that a drug can inhibit cellular invasion through affecting biomechanical properties of cancer cells [62]. Ech affected the invasion activity of the cell lines in this report, and could present a new treatment regimen for malignant tumors [63].

For understanding the molecular mechanism regulating the relationship between the viscoelastic and tumorigenic properties in ovarian cancer cells, the microfilament density of F-actin cytoskeleton was examined by fluorescence imaging of these cells after treatment with 0.25 μM Ech for 0, 3 and 6 h (Figure 7). The changes in the F-actin cytoskeleton may contribute to the changes of tumorigenic properties in ovarian cancer cells treated with Ech. The changes of tumorigenic properties and tumor progression are accompanied by a remodeling of the cytoskeleton. Earlier predictions have supported that the viscoelastic properties of highly invasive cancer cells could be associated with a difference in the F-actin cytoskeleton [64–65]. Each of these tumorigenic transformation processes is regulated by the dynamic biomechanical behavior of the F-actin cytoskeleton within the examined ovarian cells, and the possible underlying mechanical properties were subsequently characterized [66–67].

### Correlation of viscoelastic and tumorigenic properties of ovarian cancer cells treated with Ech

**Figure 8:**
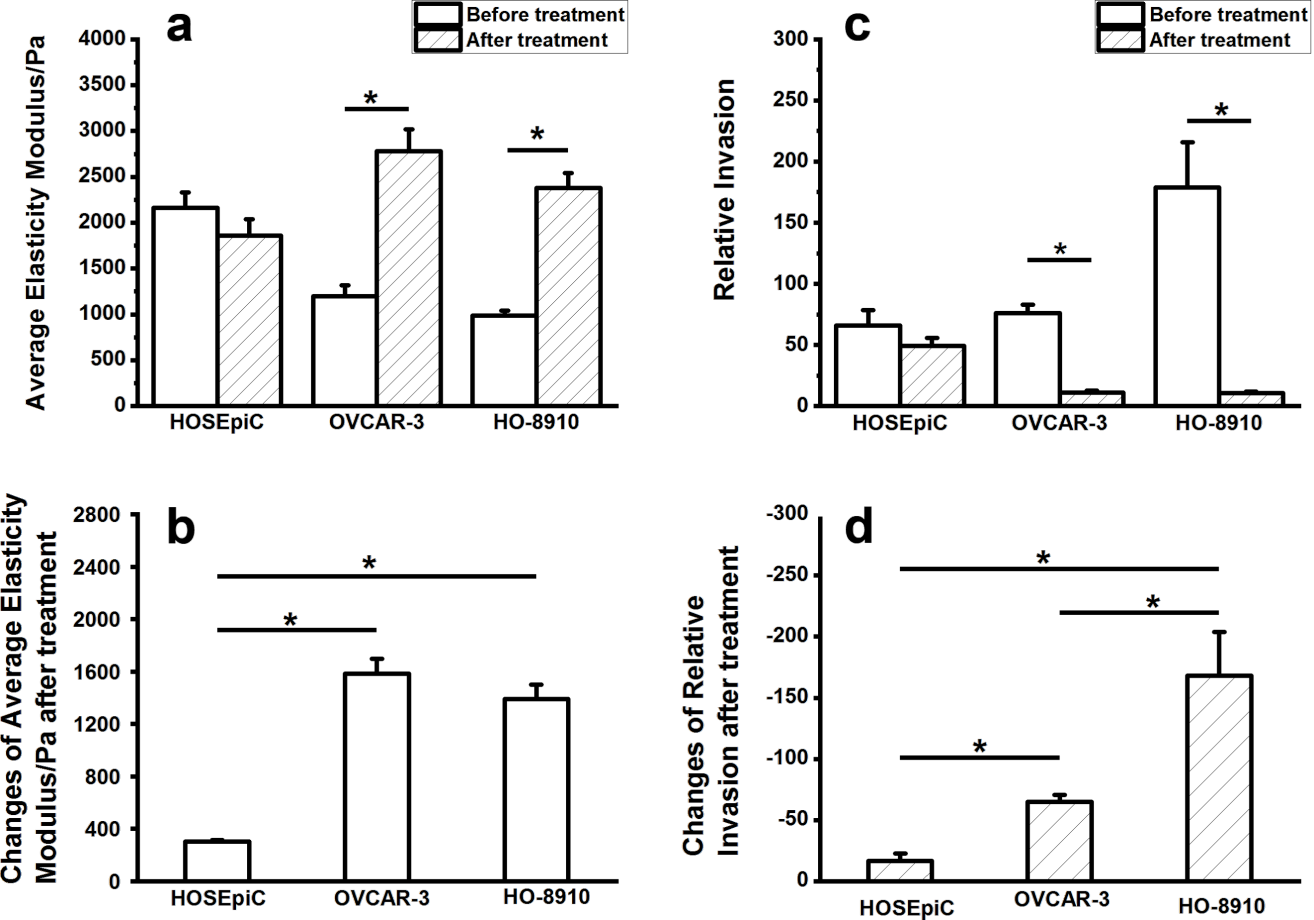
Analysis of average elasticity and invasion in ovarian cells treated with echinomycin. a: Average elasticity of ovarian cells before and after echinomycin treatment. b: Changes of the average elasticity after echinomycin treatment. c: Relative invasion of ovarian cancer cells with and without echinomycin treatment. d: Changes of the relative invasion of ovarian cancer cells after echinomycin treatment. The data are present as mean ± SE, and the asterisk indicates *p* < 0.05.

The correlation between viscoelastic and tumorigenic properties was analyzed (Table 2) and further confirmed that the elastic properties are significantly related to the migration and invasion of ovarian cancer cells (Table 2). Furthermore, the changes of average elasticity and cell invasion were analyzed after exposure to Ech (Figure 8) and the results showed no obvious changes of average elasticity (Figure 8a) and cell invasion (Figure 8c) in HOSEpiC cells, while there was a significant increase in OVCAR-3 and HO-8910 cells after Ech treatment (Figure 8a,c). Interestingly, the cell invasion of OVCAR-3 and HO-8910 cells was obviously increased compared to HOSEpiC cells (Figure 8d), which is also consistent with the changes of average elasticity among these cells (Figure 8b). The detailed mechanism needs to be investigated further in the future.

**Table 2:** Correlation analysis of elasticity and migration, invasion and F-actin density after exposure to echinomycin.

Ech elasticity		HOSEpiC	OVCAR-3	HO-8910

migration	HOSEpiC	−0.936		
	OVCAR-3		−0.872	
	HO-8910			−0.910
	
invasion	HOSEpiC	−0.915		
	OVCAR-3		−0.983	
	HO-8910			−0.869
	
F-actin density	HOSEpiC	0.833		
	OVCAR-3		0.926	
	HO-8910			0.845

## Conclusion

To our knowledge, the present study examined for the first time the association of viscoelastic properties measured by using atomic force microscopy with the invasion of ovarian cancer cells. A lower viscoelasticity was found in more malignant ovarian cancer cells. Also the migratory and invasive potential of the cancer cells increased with decreased cell viscoelasticity. Furthermore, the results of the treatment with the anticancer compound echinomycin suggested that the association of cell elasticity with the invasion of ovarian cancer cells may be caused by differences in the F-actin cytoskeleton. The present study not only provides a new method to investigate the invasive mechanisms of ovarian cancer cells, but also showed that AFM might be an effective analytical approach during very early diagnosis of cancer at the level of single living cells.

## Experimental

### Cell culture

Three ovarian cell lines, HOSEpiC (human ovarian epithelial cell line, BeNa Culture Collection, Beijing, China), OVCAR-3 (human cancerous ovarian cell line, BeNa Culture Collection, Beijing, China) and HO-8910 (human cancerous ovarian cell line, BeNa Culture Collection, Beijing, China), were purchased and used. HOSEpiC and HO-8910 cells were cultured in RPMI 1640 medium supplemented with 10% fetal bovine serum (FBS) and 1% penicillin/streptomycin solution, while OVCAR-3 cells were grown in RPMI 1640 with 20% FBS, 0.01 mg/mL bovine insulin and 1% penicillin/streptomycin solution. These cells were incubated and cultured at 37 °C in a humidified atmosphere of 5% CO_2_. For AFM experiments, the ovarian cancer cells were seeded in 35 mm culture dishes with a density of 1 × 10^4^ mL^−1^ for 24 h. Different concentrations of echinomycin were added into the culture dishes for 3 h of treatment. After the treatments, cells were then washed twice with PBS and immediately used for AFM measurements in 2 mL medium.

### Viscoelastic measurement

The viscoelasticity of cells was measured by using AFM (Nano Wizard III, JPK, Berlin, Germany) equipped with an inverted optical microscope (Leica, Germany). The viscoelastic properties were investigated in the force spectroscopy working mode. The culture dishes were placed in a Petri dish heater (JPK instrument, Berlin Germany) and maintained at 37 °C during the AFM indentations. Force–distance curve-based AFM measurements were carried out to calculate the optical photodiode deflection sensitivity and the cantilever spring constant was verified by the thermal noise method before experiments. MLCT cantilevers (Bruker, USA) made of silicon nitride with approximate spring constant values of 0.01 N·m^−1^ were employed in all AFM experiments. Selected areas surrounding the nuclei of cells (3 μm × 3 μm) were chosen for measurements in medium at room temperature. The indentation force was 1 nN with a constant velocity of 5 μm·s^−1^. All data were analyzed using the JPK data processing software [68]. The elastic modulus was acquired based on the Hertz model, and the viscosity was calculated by the method proposed by Rebelo’s group [65].

### Cell migration assay

The cells were seeded in 6-well plates at a density of 8 × 10^6^ per well and were cultured until confluence reached approximately 95%. The confluent monolayer was wounded using a sterilized 10 μL pipette tip, and then washed three times with PBS to remove dislodged cells. The culture medium was also changed to the serum-free medium. Pictures of the area of wound closure were taken with an inverted microscope. Then, the samples were incubated at 37 °C in a humidified atmosphere of 5% CO_2_ for later analysis. Cells migrated into the surface area of wound closure and the average distance of migrating cells were monitored by collecting digitized images after designated periods of time.

### Cell invasion assay

The invasion of ovarian cancer cells was analyzed by cell culture insert (Corning, 8.0 μm pore size) coated with a PET membrane according to the instructions of the manufacturer. A total of 1 × 10^4^ cells suspended in 500 µL serum-free medium was loaded into the upper chambers, and the bottom chamber was filled with 500 μL medium containing 10% FBS to stimulate invasion. The cells were incubated for 24 h and then the invading cells in the bottom of the chamber insert were stained with Giemsa. The numbers of invading cells were calculated from photographs at five randomly selected sites. Each assay was conducted at least three times.

### Confocal imaging of the microfilament skeleton

The cytoskeletal organization in ovarian cancer cells was investigated by confocal imaging. The cells were seeded into 35 mm cover glass bottom culture dishes (Nest) at a density of 5 × 10^4^ mL^−1^ and cultured in the 37 °C incubator for 2 days prior to staining. Then, the culture medium was removed and 1 mL PBS was added to each culture dish. After washing three times with PBS, the samples with or without echinomycin treatments were fixed by 4% paraformaldehyde (PFA) for 15 min, and 0.1% Triton-X-100 was used for permeabilization. After that, the cells were stained with ActinGreen (KeyGEN BioTECH). A laser scanning confocal microscope (SP8, Leica) was used to image the cytoskeletal organization by F-actin. The fluorescence imaging was captured at 488 nm excitation wavelength. Moreover, the images were processed with the software Image J.

### Statistical analysis

The data were reported as mean ± standard error (SE). Independent samples *t*-test was used to analyze the difference between two groups. Statistical analysis was conducted using SPSS software. The value of *p* < 0.05 was considered statistically significant.

### Availability of data and materials

All data generated or analyzed during this study are included in this published article.
